# Nailfold videocapillaroscopy – a novel method for the assessment of hemodynamic incoherence on the ICU

**DOI:** 10.1186/s13054-024-05194-6

**Published:** 2024-12-03

**Authors:** Sebastian Kintrup, Lukasz Listkiewicz, Philip-Helge Arnemann, Nana-Maria Wagner

**Affiliations:** 1 Department of Anesthesiology, Intensive Care and Pain Medicine, University Hospital Münster, Münster, Germany; 2 Department of Anesthesiology, Intensive Care, Emergency and Pain Medicine, University Hospital Würzburg, Würzburg, Germany

**Keywords:** Comparison, Incident darkfield microscopy, Nailfold videocapillaroscopy, Critical illness, Microcirculation

## Abstract

**Background:**

Loss of hemodynamic coherence is a phenomenon in critically ill patients. Due to inflammatory events and endothelial remodeling, macro- and microhemodynamics are decoupled from each other, resulting in microcirculatory disturbances and end organ ischemia despite adequate vital parameters. So far, quantification of perfusion of vessels with < 100 μm diameter on the intensive care unit (ICU) was regularly performed with incident darkfield (IDF) microscopy. Nailfold videocapillaroscopy (NVC), however, is an established and easy method for visualization of the microcirculation in chronic diseases. We here evaluated NVC in critically ill patients and compared its use with consensus microcirculatory assessment of IDF-microscopy.

**Methods:**

A new score, the capillary microcirculation (CapMic) score summarizing the microcirculation of the nail fold at four regions of digitus III, IV and V in a number between 0 (= no microcirculation) and 1 (= completely preserved microcirculation) was first established in 10 healthy volunteers and compared to the Microangiopathy Evolution Score (MES) standardized for NVC in chronic diseases. Then, n = 60 critically ill patients were recruited from a surgical ICU. Consensus-defined IDF scores and NCV data were compared at a single time point.

**Results:**

Evaluation of the CapMic score in 10 healthy volunteers at rest and under iatrogenic limb ischemia showed robust changes (0.80 ± 0.03 vs. 0.51 ± 0.12, p < 0.001). In critically ill patients, the IDF microscopy parameters “proportion of perfused vessels” (PPV) and “microvascular flow index” (MFI) inversely correlated with the MES (Spearman’s R = -0.590, p < 0.001; Spearman’s R = −0.585, p < 0.001). There was a positive correlation between PPV and the CapMic score (Spearman’s R = 0.714, p < 0.001) and between MFI and the CapMic score (Spearman’s R = 0.711, p < 0.001) and an inverse correlation between MES and the CapMic score (Spearman’s R = −0.610, p < 0.001). Both sublingual and nailfold microcirculation deteriorated under rising norepinephrine- and crystalloid volume-requirements.

**Conclusion:**

NVC-imaging provides comparable information on the microcirculation in critically ill patients compared to sublingual IDF microscopy. NCV could represent a new, additional method for diagnosing microcirculatory parameters on the ICU.

## Background

In the human body, vessel diameters range from 2.5cm to 6μm. Vessels > 100μm represent the macrocirculation. In critical care medicine, this part of the patients’ circulation is routinely monitored by using systemic variables as blood pressure and cardiac output. All vessels < 100μm belong to microcirculation. These vessels further split up into terminal vascular networks consisting of arterioles, capillaries and venules with diameters < 20μm. The microcirculation has vital functions as it is responsible for organ oxygen supply, solute exchange between intravascular and interstitial space, immune defense as well as transport of hormones to their active sites [1]. Endothelial cells and smooth muscle cells, both being part of microcirculatory vessel walls, are involved in many of these processes and further regulate vasotone and permeability that, in turn, again affect the macrocirculation. The mutual interaction between macro- and microcirculation is referred to as “hemodynamic coherence” and allows conclusions to be drawn from systemic variables to microcirculatory conditions under physiological states [2].

More than 20 years ago, first data was published regarding hand-held videomicroscopes, enabling physicians to also make microcirculation visible for the very first time [3]. Nowadays, sublingual incident darkfield (IDF-)microscopy, that has been developed from the original orthogonal polarization spectral imaging, represents the gold-standard technology for performing microcirculatory imaging. In 2018, consensus guidelines on the practical use and interpretation of the sublingual microcirculation were published [4]. Due to direct microcirculatory visualization, it is now known that microvessels suffer from profound changes under conditions of systemic inflammation: Endothelial dysfunction, glycocalyx degradation, rupture of cadherins and disbalance between vasodilating and -constricting agents lead to microcirculatory impairment and decoupling of the micro- from the macrocirculation [5].

Despite its importance for evaluation of tissue perfusion and organ integrity, microcirculatory parameters are still not routinely monitored in patients with systemic inflammation such as after major surgery. One reason for the restrained use of microcirculatory assessment might be that there are currently no validated additional methods and IDF-microscopy is simply not available to a broad majority of intensivists. Therefore, we intended to evaluate a second imaging technique called “nailfold videocapillaroscopy” (NVC) that is known from the field of dermatology and rheumatology. The technique has first been used in 1922 and enables to visualize nailfold capillaries located at patients’ fingers by using a digital videocapillaroscope or, alternatively, a digital USB-microscope [6]. NVC is inexpensive, user-friendly, free of motion artifacts and images can be generated rapidly with easy access. Moreover, patients requiring non-invasive ventilation can also be examined. In modern clinical routines, NVC has only been used for diagnosis and monitoring of chronic diseases, yet: It is officially part of systemic sclerosis diagnostics as microvascular abnormalities within the nailfold capillaries are key features of the disease [7].

As the nailfold vascular bed also represents the microcirculation, we aimed to investigate whether NVC might be a suitable means for bedside microcirculatory assessment during critical illness, as well. We here performed a pilot study comparing sublingual IDF-microscopy with NVC in 60 intensive care unit patients and correlating variables of both procedures.

## Materials and methods

### Patient selections

From July 2023 to April 2024, we recruited *n* = 60 adult patients on the intensive care units (ICU) of the University Hospital Münster. Written informed consent was obtained from patients or their legal representatives. The study was approved by the Ethics Committee of the University Hospital Münster (ID: 2023–203-f-S). Exclusion criteria were peripheral artery disease, thrombosis of the upper extremity, finger amputations and surgery within the oral cavity. Patients were deliberately heterogeneously selected with various diagnoses like sepsis, traumatic intracerebral hemorrhage, following cardiac and abdominal surgery, or bleeding from esophageal varices. Due to the variability of pathologies, hemodynamic states were also heterogenous ranging from no catecholamine dependence to significant support by vasopressors and inotropic agents. All patients were sedated and on mechanical ventilation to ensure data acquisition without any movement artifacts. At a single point of time, both sublingual and nailfold microcirculation were assessed using IDF-microscopy and NVC. Measured values from both procedures were correlated with each other.

### IDF-microscopy

Sublingual microcirculation was measured with IDF-microscopy using a hand-held video microscopy camera (CytoCam, Braedius Medical B.V.). At least three recordings per patient, originating from different sublingual regions, were obtained by gently putting the videomicroscope on the sublingual mucosa without causing pressure artifacts. According to the consensus guidelines on the assessment of the sublingual microcirculation, each recording was motion-free, evenly illuminated, focused and had a length of 5 s [4]. Furthermore, saved recordings were software-corrected using “CytoCamTools V4” to minimize image movement. Video analysis was performed manually using the software “Capillary Mapper 1.4.5”. All recordings were obtained and analyzed by the same experienced investigator. Primary variables assessed were “proportion of perfused vessels” (PPV) and the “microvascular flow index” (MFI).

#### Nailfold videocapillaroscopy (NVC) and microangiopathy evolution score (MES)

**Fig. 1:**
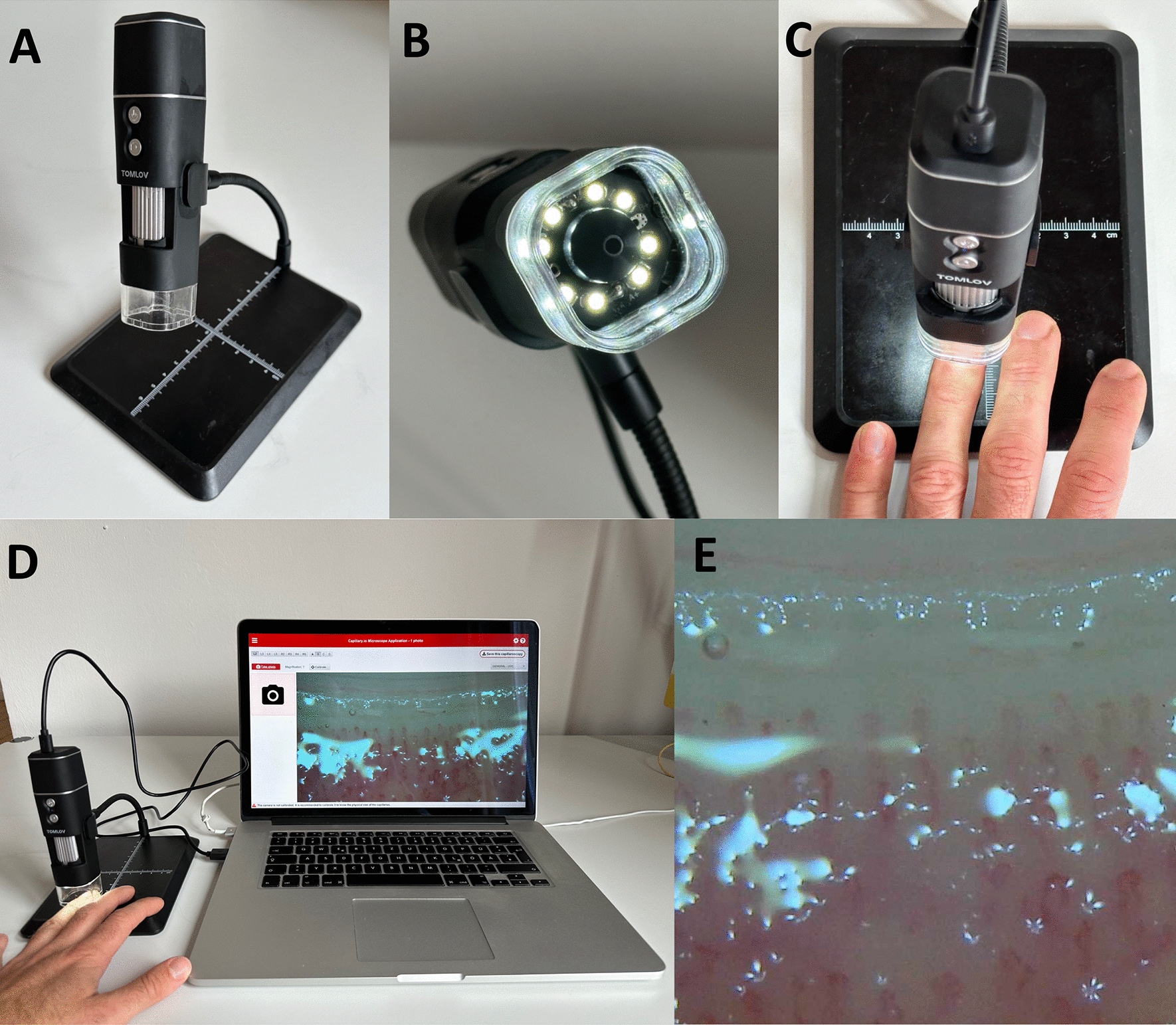
Technical device for NVC-imaging. **A** TOMLOV Wi-Fi Digital Microscope, Model DM1. **B** Camera lense and LED-spots. **C** Positioning of a finger under the device after dropping one drop of walnut oil on the nailfold area. **D** Overview of the examination set. **E** Nailfold microcirculation

**Fig. 2:**
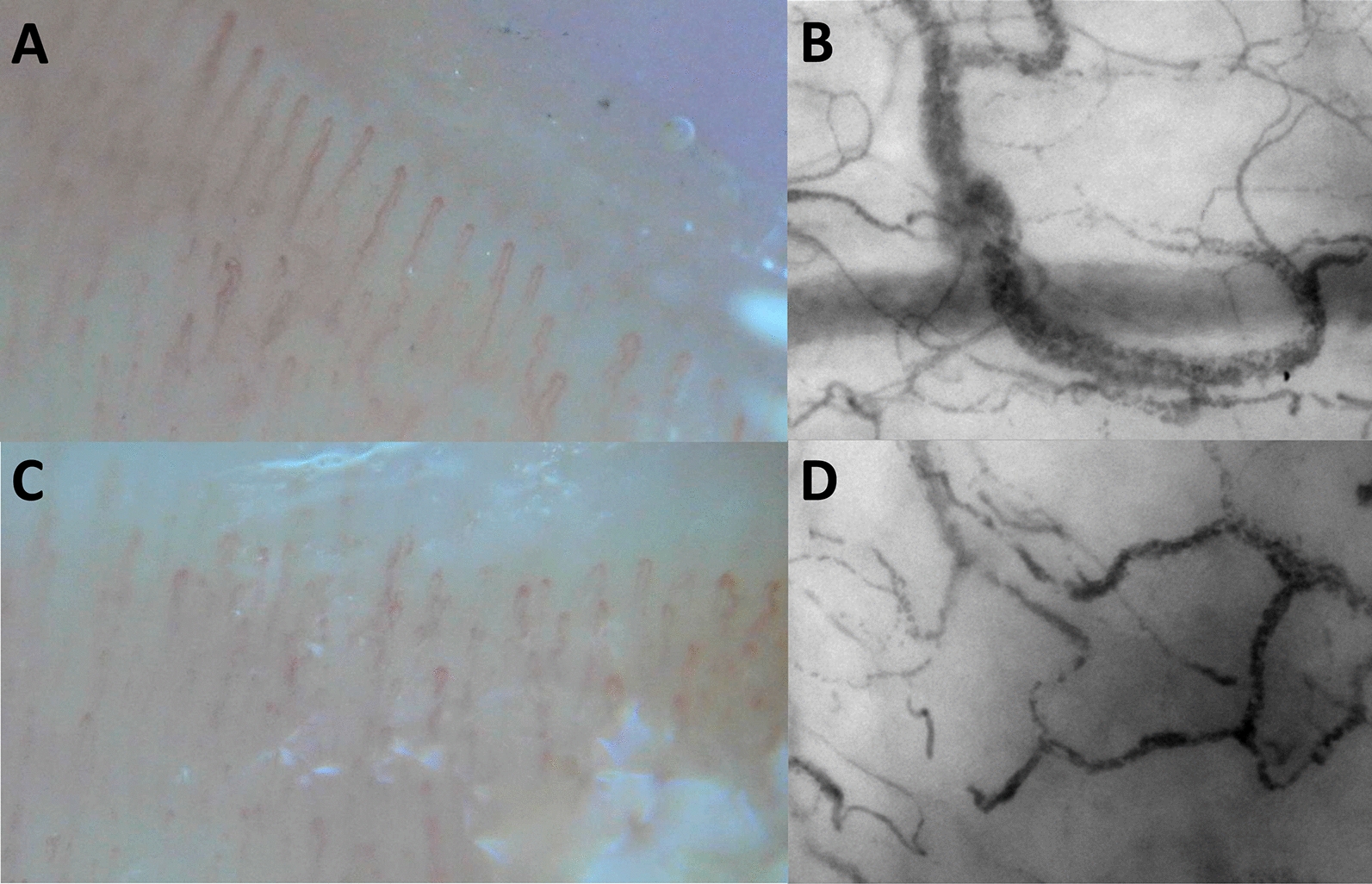
Examples of NVC- and IDF-imaging. **A** Patient with well perfused nailfold capillaries. **B** Corresponding sublingual capillaries. **C** Patient with poorly perfused nailfold capillaries. **D** Corresponding sublingual capillaries

Nailfold microcirculation was measured using an USB digital microscope (TOMLOV Wi-Fi Digital Microscope, Model DM1, Fig. 1). Ahead of the examination it was ensured that the patients’ hand was positioned without tension or pressure. One drop of walnut oil was placed on the nailbeds of finger III, IV and V of one upper extremity. Nailfold capillaries were then displayed and documented by connecting the microscope to a laptop in the following 4 fields: Left lateral, left paramedian, right paramedian and right lateral. In each position, a 50 × magnification was first used to find an adequate area and a 200 × magnification with 1 mm diameter was used to take images for quantification (Fig. 2). Therefore, 12 images of the nailfold microcirculation per patient were drawn. For microcirculatory quantification, two scoring systems were applied. The first scoring system was the “microangiopathy evolution score” (MES) [8]. This score has been used since 2008 for diagnostics in systemic sclerosis and represents an official tool mentioned in the ACR/EULAR classification [9]. MES focuses on assessment of the following microvascular abnormalities associated with systemic sclerosis: Presence of enlarged or giant capillaries, microhemorrhage, loss of capillaries, disorganization of the vascular bed and capillary ramification. Applying a semiquantitative rating scale, each capillary abnormality was scored in each of the 4 consecutive fields depending on its occurrence (0 = no changes, 1 = less than 33% of capillary alterations/reductions, 3 = more than 66% of capillary alterations/reductions per linear millimeter) [8]. The sum of all microvascular abnormalities per finger was determined and a mean value per finger was established. Final MES was formed by adding the mean values of three examined fingers and building a general mean value per patient.

#### Nailfold videocapillaroscopy (NVC) and CapMic score

**Fig. 3:**
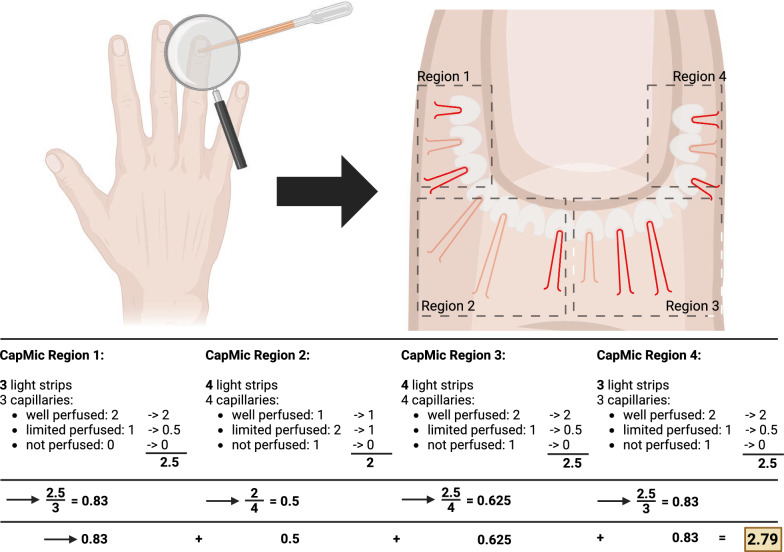
Structure of the CapMic score. The third, fourth and fifth finger are investigated according to the following procedure: One drop of walnut oil is put on the nailfold. A 200 × magnification is used to depict light strips with associated capillaries in the following 4 regions: Left lateral, left mediolateral, right mediolateral, right lateral. The number of light strips per region is counted. Each associated capillary is scored from 0 (invisible and not perfused) to 1 (fully perfused). Ratio between capillary perfusion and number of light strips per region is calculated. The 4 ratios per finger are summarized to a mean value. To calculate the final CapMic score, the 3 mean values per finger are summarized again

The second scoring system represents a newly developed method for assessment of nailfold perfusion we termed ‘capillary microcirculation score (CapMic score)’. Quantification was performed from identical images compared to MES-scoring. CapMic scoring is based on the following considerations: In NVC, each capillary is surrounded by a light strip which represents the transition area from epidermal stratum granulosum to dermal stratum papillare. In case of malperfusion, light strips can still be seen even if the associated capillary might be undetectable. Due to this circumstance, the examiner knows the number of capillaries one would actually expect to be detectable. Scoring was performed as follows: In each field of the 3 examined fingers, the number of light strips was counted. The perfusion of each capillary contained was graded (0 = no capillary detectable, 0.5 = malperfused capillary, 1 = fully perfused capillary). The sum of perfusion grading was divided by the number of visible light strips, generating a score ranging from 0 (no perfusion) to 1 (full perfusion) for each field of each finger. In the following, mean values were calculated for each finger and from these, a mean value for each patient was generated representing the CapMic score (Fig. 3). As the CapMic score is novel, we aimed to validate if nailfold capillary perfusion in NVC-imaging is affected by major hemodynamic changes ahead of patient recruitment. For this purpose, NVC was first performed in *n* = 10 healthy volunteers. In each volunteer, two examinations were performed. The first examination took place under resting conditions. The second examination was performed under iatrogenic ischemia of the extremity. To achieve this, the outstretched limb was first held upwards for 10 s. A blood pressure cuff on the upper arm was inflated to suprasystolic values. Then, the arm was positioned without any tension and pressure and NVC was performed for a second time. The CapMic score values between the first and second examination were compared.

### Statistical analyzes

Data are presented as mean values with standard error of mean. The Shapiro–Wilk-test was used to test if values were normally distributed. For correlation analysis, Pearson- and Spearman-tests were used depending on data distribution. The student’s t-test and the Mann–Whitney-Test were used to determine significance level. A p-value < 0.05 was considered statistically significant. Categorial variables were analyzed using cross tables and the chi square test. All analyzes were performed with Prism 10 and SPSS Statistics 14.

## Results

### The CapMic score sufficiently detects microcirculatory impairment

**Fig. 4:**
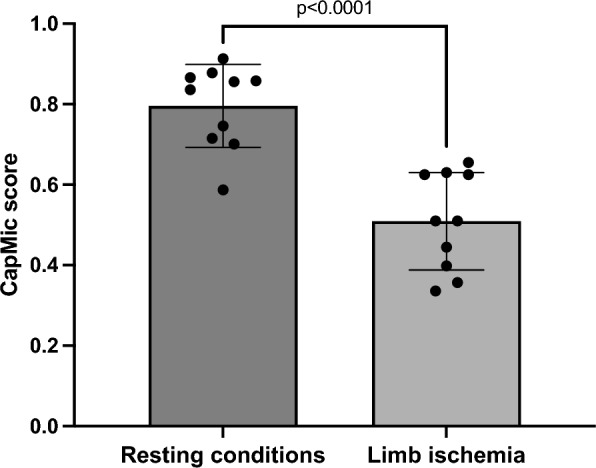
Validation of the CapMic score. Nailfold microcirculation was significantly higher under resting conditions compared to limb ischemia

We first validated the CapMic score in *n* = 10 healthy volunteers ahead of patient recruitment. Under resting conditions, the CapMic score was 0.80 ± 0.03. After temporary induction of limb ischemia, the score decreased to 0.51 ± 0.12, p < 0.001. Importantly, also the Microangiopathy Evolution Score (MES) was susceptible to acute changes in limb perfusion: Whereas MES was 0.19 ± 0.05 under resting conditions, the score increased to 0.27 ± 0.05 (p = 0.033) during circulatory impairment (Fig. 4).

### IDF parameters correlate with NVC parameters

*N* = 60 ICU-patients were included in the study. 71.7% were male, 28.3% were female. Mean age was 65.5 ± 1.7 years. Patients had been admitted to the ICU for 4.5 ± 1.3 days ahead of study recruitment. All patients were mechanically ventilated. 83.3% needed catecholamine therapy and 13.3% were dependent on continuous renal replacement therapy (CRRT). Further baseline criteria are depicted in Table 1.

**Table 1:** Patient data on demographics, comorbidities, length of hospital stay and clinical data at the time of microcirculatory assessment

*Demographic data*	
Age (years)	65.5 ± 1.7
Sex (%)	Male: 72, female: 28
Weight (kg)	81.4 ± 2.3
Height (cm)	175 ± 1.1
Body mass index (kg/m^2^)	26.6 ± 0.7
*Comorbidities*	
Arterial hypertension (%)	37 (62)
Coronary heart disease (%)	24 (40)
Congestive heart failure (%)	13 (22)
Peripheral arterial occlusive disease (%)	3 (5)
Atrial fibrillation (%)	20 (33)
Asthma bronchiale (%)	1 (2)
Chronic obstructive pulmonary disease (%)	7 (12)
Liver insufficiency (%)	5 (8)
Chronic kidney disease (%)	10 (17)
Diabetes mellitus (%)	16 (27)
*Length of stay*	
Neurological disorders (%)	16 (27)
Length of ICU stay (d)	4.5 ± 1.3
Length of hospital stay (d)	5.6 ± 1.4
*Clinical data at the time of microcirculatory assessment*	
Glasgow coma scale	5.9 ± 5.6
Horowitz-index (paO_2_/fiO_2_)	2.7 ± 0.2
MAD (mmHg)	77.8 ± 1.6
Norepinephrine (μg/kg/min)	0.1 ± 0.02
Vasopressin (IE/h)	0.5 ± 0.1
Epinephrine (μg/kg/min)	0.01 ± 0.0
Dobutamine (μg/kg/min)	1.3 ± 0.3
Bilirubin (mg/dL)	1.3 ± 0.2
Creatinine (mg/dL)	1.2 ± 0.1
Platelets (1000/mL)	170.6 ± 11.5
HbA1c of diabetic patients (%)	6.7 ± 1.2

**Fig. 5:**
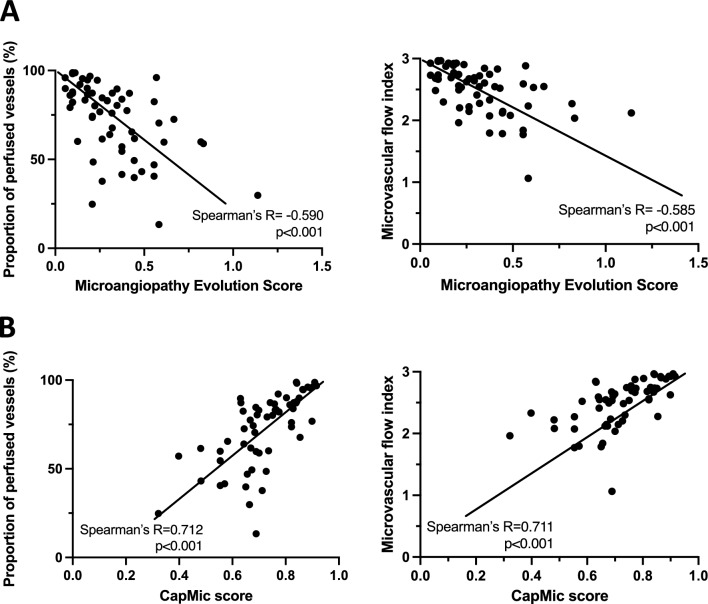
Correlation of IDF-imaging with NVC-imaging. **A**: Correlation of MES with PPV and MFI. **B**: Correlation of CapMic score with PPV and MFI. Statistics: Spearman correlation

In all patients, proportion of perfused vessels (PPV) and the microvascular flow index (MFI) were assessed as sublingual microcirculatory parameters whereas MES and CapMic score served as parameters for nailfold microcirculation. As MES represents a validated scoring system we first aimed at investigating if MES-values correlate with PPV and MFI. Spearman-correlation showed a negative correlation between MES and PPV (Spearman’s R = -0.590, p < 0.001) as well as for MES and MFI (Spearman’s R = -0.585, p < 0.001) (Fig. 5A). In the following, the CapMic score was tested for correlation with sublingual parameters. There were positive correlations between CapMic score and PPV (Spearman’s R = 0.714, p < 0.001) as well as between CapMic score and MFI (Spearman’s R = 0.711, p < 0.001) (Fig. 5B). Finally, MES and CapMics score also negatively correlated with each other (Spearman’s R = -0.610, p < 0.001). The lower IDF-parameters were the greater individual MES- but also CapMic score-values scattered.

### IDF- and NVC parameters correlate with variables of macrohemodynamic resuscitation

**Fig. 6:**
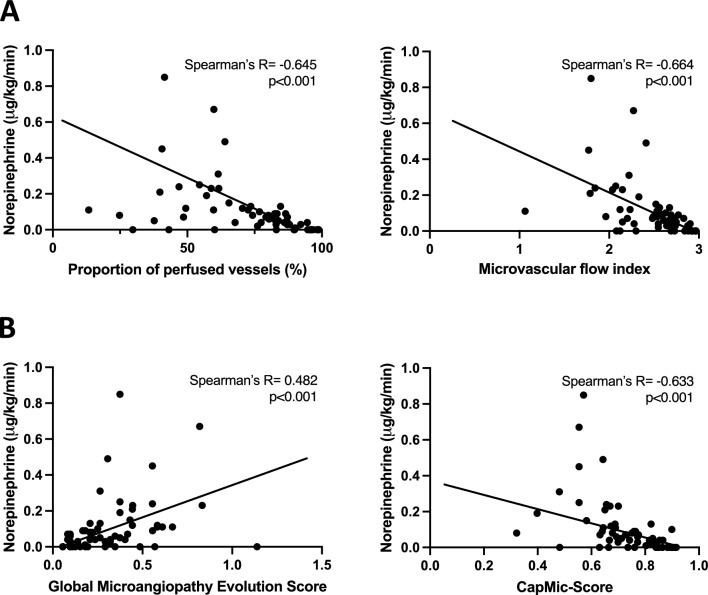
Correlation of norepinephrine-requirements with microcirculatory parameters. **A**: Correlation of norepinephrine with PPV and MFI. **B**: Correlation of norepinephrine-requirements with MES and CapMic score. Statistics: Spearman correlation

In the following, we analyzed whether hemodynamic conditions are reflected by microcirculatory parameters. Both PPV and MFI inversely correlated with norepinephrine-requirements at the time of microcirculatory measurements (Spearman’s R = -0.645, p < 0.001; Spearman’s R = -0.664, p < 0.001) (Fig. 6A). NVC-parameters showed correlations with norepinephrine-requirements, as well (MES: Spearman’s R = 0.482, p < 0.001; CapMic: Spearman’s R = -0.633, p < 0.001) (Fig. 6B). The worse both SLM- and NVC-parameters were the greater the individual differences regarding norepinephrine requirements became.

**Fig. 7:**
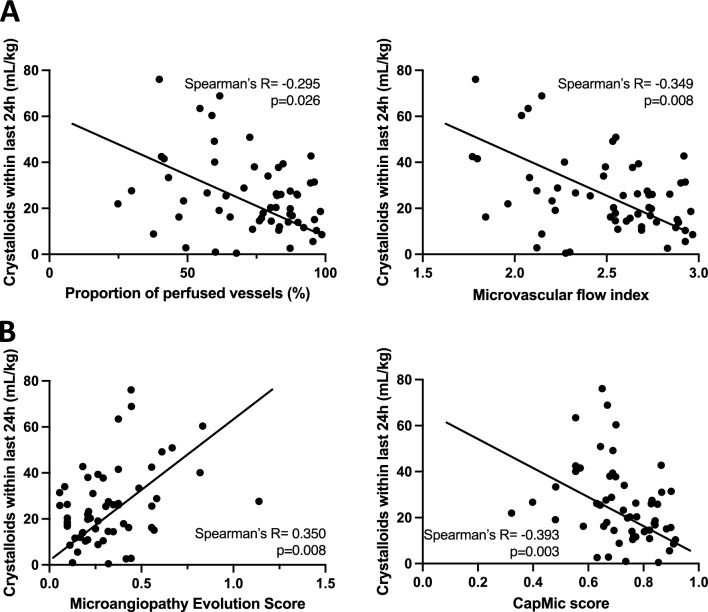
Correlation of administered crystalloid volume within last 24h with microcirculatory parameters. **A**: Correlation of crystalloid volume with PPV and MFI. **B**: Correlation of crystalloid volume with MES and CapMic score. Statistics: Spearman correlation

Further data revealed that the higher PPV- and MFI-values were, the lower was patients’ need for crystalloid volume over the last 24h (PPV: Spearman’s R = -0.295, p = 0.026; MFI: Spearman’s R = -0.349, p = 0.008) (Fig. 7A). Equal observations were made for NVC-parameters MES and CapMic score (MES: Spearman’s R = 0.393, p = 0.003; CapMic score: Spearman’s R = 0.350, p = 0.008) (Fig. 7B). Again, scattering was dependent on the extent of microcirculatory impairment.

## Discussion

Critical illness is often accompanied by inflammation and states of shock. Resuscitation from these life-threatening conditions regularly relies on the improvement of macrohemodynamic parameters, derived from invasive blood pressure measurement and extended hemodynamic monitoring. Lastly, crystalloid volume and/or vasoactive agents are administered to enable red blood cells to adequately supply organs with oxygen. However, it is mostly uncertain whether this therapeutic aim is really achieved even if macrohemodynamics seem to improve: As arterioles, venules and capillaries suffer from endothelial remodeling, leading to vasoconstriction, procoagulation, breakdown of adaptor-proteins and extravasation of fluids, it cannot be ruled out that administered agents possibly have no causal effect [10].

One possibility to confirm effectiveness of resuscitation efforts would be to regularly quantify microcirculation by performing incident darkfield (IDF-) microscopy. Recently, it has been proven that sublingual microcirculation is indeed significantly altered under postoperative, inflammatory conditions [11]. Unfortunately, IDF-imaging is still barely part of clinical routines as it is not ubiquitously available. We here presented the additional imaging technique of nailfold videocapillaroscopy (NVC) for judging microcirculation during critical illness.

So far, NVC-imaging has mostly been used for the relation of morphologic vessel irregularities to chronic diseases. If nailfold capillaries are also capable to reflect (semi-)acute changes in microcirculation has never been quantitatively assessed. Our proof-of-principle trial examining 10 healthy volunteers showed that nailfold capillaries indeed change their appearance under sudden limb ischemia: The predominant effect was a loss of capillary density. However, some morphologic irregularities were also detectable. The fact that loss of capillary density appears to be—at least under artificial ischemic conditions—the predominant reaction, puts even more emphasis on the CapMic score-development as this score focuses on the number of visible capillaries rather than on morphologic properties. Conversely, this finding suggests that the microangiopathy evolution score (MES) is probably not the ideal score for assessment of nailfold microcirculation in the context of critical illness.

Next, it was uncertain if parameters for sublingual microcirculation correlate with nailfold-derived scores. We found significant correlations between all four parameters assessed. However, accuracy decreased with deteriorating microcirculatory conditions. This either indicates different baseline perfusion of the capillary beds even ahead of the critical illness or that sublingual and nailfold microcirculation do not react completely equal to disease-associated stimuli in certain patients: Some stimuli might reduce sublingual microcirculation to a greater extent than nailfold microcirculation and vice versa. So far, IDF- and NVC-imaging had been compared once in the field of systemic sclerosis. Here, the sublingual “red blood cell fraction” and the perfused boundary region score were compared to nailfold MES and significant correlations between the two imaging techniques were found. Particularly, a loss of capillary density for systemic sclerosis patients was described [12]. This finding proves that nailfold capillaries at least react similar to conditions of inflammation and shock compared to sublingual capillaries.

Furthermore, the NVC-technique had been judged as inappropriate for critically ill patients due to the nailfold area’s high susceptibility to body temperature changes and consequent nailfold capillary vasodilation or –constriction [13]. Our study, however, indicates that NVC-imaging might be suitable for the quantification of nailfold microcirculation in the context of acute, severe illness: First, PPV and MFI, already validated for this purpose and not significantly affected by body temperature, correlated with NVC-parameters. Second, analyzes of patients’ body temperature revealed a range lasting from 35.7–39.1°C, the mean body temperature was normothermic, i.e. 37.1°C. Body temperature did not correlate with NVC-parameters as we confirmed in an additional analyzes. Thus, it is likely that the effect of body temperature on NVC-imaging is negligible as long as the temperature lies within normo- or hyperthermic regions and ambient temperature is constant. Especially for young patients between 20–39 years, however, it is proven that coldness to the fingertip reduces capillary density [14]. The fact that only 4 patients aged < 39 years were included in our study might be an explanation for the reduced influence of temperature to our NVC-measurements.

Interestingly, habits and lifestyle also influence nailfold microcirculation. It was found that healthy lifestyle including sports, sufficient sleep and abstaining from alcohol and smoking leads to straighter and longer capillary loops whereas twisted, brushy and small loops indicated unhealthy lifestyle [14] – all these criteria did neither affect MES nor CapMic score. However, unhealthy lifestyle also led to loss of capillary density. This, however, could have been misinterpreted as a lack of capillary perfusion which represents a limitation of our study. Furthermore, it is important to bear in mind that nailfold capillaries – in contrast to sublingual vessels – will also always reflect chronic disease states like hypercholesterinemia, type 2 diabetes mellitus, pulmonary arterial hypertension or various rheumatic disorders which might cause misinterpretations when evaluating microcirculation [15–18].

The comparison of both IDF- and NVC-parameters with clinical characteristics revealed an inverse correlation between microcirculation and norepinephrine-doses. This finding raises doubts whether α1-adrenoceptor-agonists are suitable means to improve microcirculation during critical illness. Literature is inconsistent concerning this question: Some studies confirm improvement of sublingual microcirculation under norepinephrine-administration [19] while others report no significant effects [20]. Our study is one of the first of its kind reporting of a dose-dependent deterioration of sublingual microcirculation. Of course, reasons for this finding may also lie within the nature of patients’ critical illness which simply required certain doses of vasopressor. Regarding nailfold capillary reactions to norepinephrine, it has been reported that the nailfold capillary bed clearly reacts to the drug by constricting [21]. This pattern might represent a limitation of the technique in critically ill patients requiring vasopressors compared to less vulnerable IDF-imaging.

The more crystalloid agents patients had received over 24h, the more compromised the sublingual and nailfold microcirculation was. This matches with findings from ovine hemorrhagic shock models indicating that crystalloid solutions—instead of colloids—only improve the macro- but not the microcirculation [22]. As crystalloid fluids lead to release of atrial natriuretic peptides and shedding of endothelial glycocalyx-structures, an associated impairment of microcirculation is well conceivable [23].

Our study has several limitations. First, it has a cross-sectional character and data was assessed at a single point of time. Second, both MES and CapMic score are unvalidated tools for use in intensive care medicine. Third, the majority of patients were male and had received open heart surgery. This, however, often correlates with unhealthy lifestyle conditions, negatively affecting capillary density. Fourth, nailfold microcirculation is to a certain extent susceptible to coldness, vasopressors and systemic, chronic diseases which possibly limits the significance of the procedure.

## Conclusion

NVC-imaging is a suitable means to assess the microcirculation in critically ill patients. NVC-parameters show significant correlations with established IDF-imaging-parameters and match up with clinical parameters like norepinephrine- and crystalloid volume-requirements. It could represent an additional method for microcirculatory assessment on the ICU, bearing possible limitations in mind.

## Data Availability

No datasets were generated or analysed during the current study.

## Abbreviations

IDF-microscopy: Incident darkfield microscopy
MES: Microangiopathy evolution score
MFI: Microvascular flow index
NVC: Nailfold videocapillaroscopy
PPV: Proportion of perfused vessels
